# Deep Lateral Wall Partial Rim-Sparing Orbital Decompression with Ultrasonic Bone Removal for Treatment of Thyroid-Related Orbitopathy

**DOI:** 10.1155/2019/9478512

**Published:** 2019-12-02

**Authors:** Álvaro Bengoa-González, Alicia Galindo-Ferreiro, Enrique Mencía-Gutiérrez, Hortensia Sánchez-Tocino, Agustín Martín-Clavijo, María-Dolores Lago-Llinás

**Affiliations:** ^1^Ophthalmology Department, 12 de Octubre Hospital, Complutense University, 28041 Madrid, Spain; ^2^Ophthalmology Department, Rio Hortega Hospital, Valladolid University, 47012 Valladolid, Spain; ^3^Dermatology Department, Queen Elizabeth Hospital, Birmingham University, B15 2TH Birmingham, UK

## Abstract

**Purpose:**

To describe the results of thyroid-related orbitopathy (TRO) treated by ultrasonic deep lateral wall bony decompression with partial rim sparing (DLW-PRS).

**Methods:**

A review was carried out, from January 2015 to September 2017, of all patients treated with ultrasonic DLW-PRS decompression using a SONOPET® (Stryker, Kalamazoo, MI, USA) ultrasonic aspirator, using a lateral, small triangle flap incision for access. The primary outcome was the change in proptosis (measured by the difference in Hertel exophthalmometry measurements). Other secondary outcomes were changes in visual acuity (VA) (using Snellen scale, decimal fraction), presence of lagophthalmos, eyelid retraction (measured by upper eyelid margin distance to the corneal reflex (MRD_1_) and lower eyelid margin distance to the corneal reflex (MRD_2_), and presence of exposure keratopathy).

**Results:**

A total of 58 orbital decompressions in 35 patients were reviewed, with 23 patients (65.7%) having bilateral decompressions. There was a female preponderance with 26 patients (74.2%), and the mean age ± standard deviation was 52.6 ± 13.9 years. Mean proptosis was 24.51 ± 1.76 mm preoperatively, reduced to 19.61 ± 1.27 mm in final follow-up. The mean reduction was 4.9 ± 1.54 mm. VA improved from 0.8 ± 0.14 to 0.9 ± 0.12, *p*=0.039. 5 of 13 patients (38.4%) with preoperative diplopia reported improvement or complete resolution after surgery. MRD_1_ was reduced from 5.25 ± 0.88 mm to 4.49 ± 0.7 mm. MRD_2_ was also reduced from 6.3 ± 0.88 mm to 5.0 ± 0.17 mm. Presence of lagophthalmos was reduced from 35 eyes (60.3%) to five (8.6%); the presence of epiphora was also reduced from 20 patients (57.1%) to 3 (8.5%) following decompression. Complications of the surgery included zygomatic hypoaesthesia in 14 (40%) patients in the early postoperative period and chewing alterations in 10 (28.5%) of the patients. All of these complications were resolved at the 6-month follow-up visit. We noted no surgical complications such as ocular or soft tissue damage, infection, inflammation, or visual loss.

**Conclusions:**

The SONOPET® ultrasonic bone curette can be used safely and effectively for DLW orbital decompression surgery. The main benefits were good visualization and handling of tissues and speed and ease of use of the equipment. This trial is registered with ClinicalTrials.gov identifier: NCT04025034.

## 1. Introduction

Thyroid-related orbitopathy (TRO), also known as Graves orbitopathy, is the most common inflammatory orbitopathy. It can present with eyelid retraction, proptosis, restrictive strabismus, and exposure keratopathy. Optic neuropathy can be sight-threatening and can have a serious impact on the patient's quality of life [1–4]. Orbital decompression has been the mainstay in the treatment of TRO for patients with compressive optic neuropathy or exposure keratopathy as well as for cosmetic rehabilitation in patients with disfiguring exophthalmos [5]. The goal of bone decompression surgery is to provide more space for the orbital contents, thus reducing proptosis, orbital pressure, pain, and discomfort [6]. Various techniques and approaches have been described for deep lateral wall (DLW). Bony decompression of the orbit, including coronal approach [7], superior crease [8–12], canthotomy [13, 14], transconjunctival “swinging eyelid” [15], and lateral triangle flap technique [16]. It can be described more simply, when the procedure is performed by an ab interno approach, removing [9] or not removing [11, 17, 18] the orbital rim or ab externo with [5, 12, 19–21] or without rim sparing [10, 21].

The effectiveness of DLW decompression is largely dependent on bone removal from the greater sphenoid wing and the frontal bone [7, 18], which is typically performed with a high-speed drill. However, the drill can be difficult to operate safely within the orbital constraints and can sometimes cause intraoperative complications such as trauma to orbital soft tissues or damage to the dura mater [19].

The SONOPET® (Stryker, Kalamazoo, MI, USA) ultrasonic aspirator is a surgical tool that uses low-frequency ultrasonic vibrations to fragment tissue while simultaneously irrigating and aspirating the surgical field. This technology is becoming widely used in orbital, lacrimal, neurological, and skull base surgery [22].

There are only a few authors who have reported partial rim sparing (PRS) using a high- speed drill [20] or high-speed drill and rongeurs [5, 23]. The first report of ultrasonic bone removal in the orbit was performed by Sivak-Callcott et al. [24] who used the SONOPET® in 13 lateral orbital decompressions and 6 external dacryocystorhinostomies. Cho et al. [19] reported a further 18 orbital decompressions in 2010. They reported that ultrasonic bone removal appeared to be superior to the standard drills and rongeurs in terms of visualization, manipulation, speed, and ease of use.

In this paper, we describe our experience using DLW- PRS decompression with a bone-cutting ultrasonic aspirator that can be customized for variable decompression of the orbit by tailoring the amount of bone removed from each wall. To our knowledge, there are only 3 papers published regarding orbital decompression using ultrasounds; ours is the largest series to report surgical outcomes using the SONOPET® in DLW decompression, removed en bloc with partial rim sparing.

## 2. Materials and Methods

This study was a retrospective, noncomparative case series. The review was carried out from January 2015 to September 2017 of all patients which had a triangle flap single-incision, ultrasound DLW-PRS decompression by a single senior surgeon (A.B-G). This study adhered to the tenets of the Declaration of Helsinki. Institutional review board approval was obtained to perform this retrospective chart analysis. All the patients signed a specific informed consent and also gave consent to be photographed. The study has been registered at ClinialTrials.gov identifier: NCT04025034.

### 2.1. Inclusion Criteria

All patients had at least a 2-year history of Graves's orbitopathy and were in the inactive phase with stable clinical activity scores for at least 12 months and had been biochemically euthyroid for more than 6 months [25]. Everyone had an exophthalmos in exophthalmometry >20 mm (for women) and >21 mm (for men) at presentation [26] (using Hertel's exophthalmometer) before they underwent surgery for rehabilitation of disfiguring exophthalmos. All patients had a minimum follow-up of 6 months. During the study period, other forms of orbital decompression for dysthyroid ophthalmopathy were performed but to properly evaluate the results of lateral decompression alone, cases in which more than one wall was decompressed were excluded. Patients with other concurrent orbital conditions were excluded.

### 2.2. SONOPET® Ultrasonic Aspirator

The SONOPET® ultrasonic aspirator consists of an ultrasonic handpiece that is connected to a base control module. The unit is foot-pedal controlled. The base module houses the controls to regulate the irrigation rate (between 3 and 40 mL/min), aspiration, and ultrasound power parameters of the machine. The power setting is expressed as a percentage of that maximum. Aspiration reaches 500 mmHg, and the aspiration setting on the machine is also expressed as a percentage of that maximum. The irrigation rate is expressed in milliliters per minute [22].

Aspiration occurs through an opening at the distal aspect of the handpiece tip, and the irrigation fluid (normal saline at 20°C) flows through a white irrigation sleeve surrounding the handpiece tip. The handpiece oscillates in a nonrotational fashion up to 25,000 times per second with a 0.36 mm width variation. The SONOPET®'s primary mechanism of action is torsional oscillation of a metal bone rasp at 25 kHz. This frequency is ideal for bone removal [27], as the microenvironment created only cuts mineralized tissue, while soft tissues are best cut at frequencies ≥34 kHz [28]. The universal handpiece fits multiple interchangeable tips that have varying lengths, sizes, and shapes designed for specific soft tissue or bone removal purposes [27]. Different sizes and angles for the cutting surface are also available.

**Figure 1:**
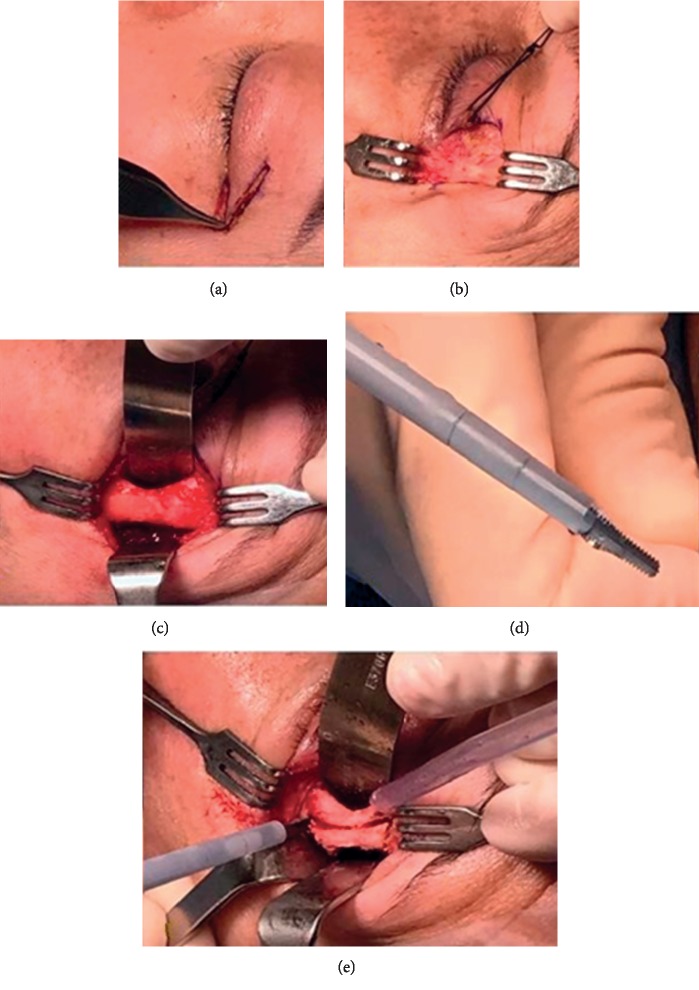
The triangular incision along the eyelid crease (a) allows excellent exposure of the orbital rim (b). The exposed lateral wall (c). The serrated aggressive knife used for osteotomies (d). The plastic irrigation sleeve on the ultrasonic tip prevents thermal damage to the skin and soft tissues. Full-thickness osteotomy made 5 mm posterior and parallel to the lateral orbital rim with the aggressive serrated knife tip, extending from the level of the orbital roof to the floor and a second full-thickness back-cut placed above the zygomatic arch (e).

**Figure 2:**
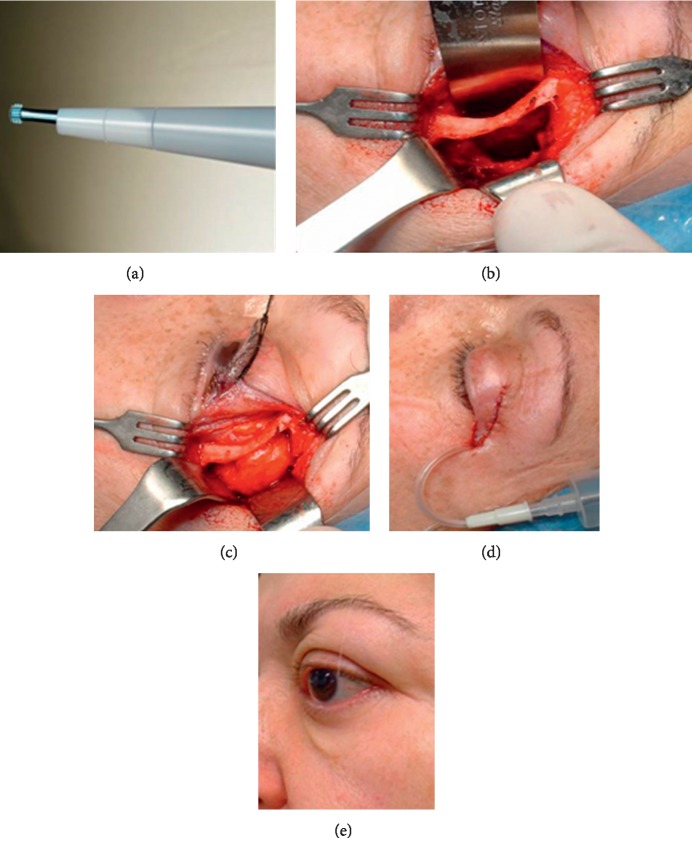
(a) Superlong Payner 360° used for trigone removal. (b) The deep lateral wall to the trigone is removed. The image shows the intact orbital rim and bony window following osteotomy. (c) Prolapse of the lacrimal gland and orbital fat in the newly opened 623 bony spaces. (d) The incision is closed in layers and vacuum drain inserted. (e) 1 year after surgery, the scar is almost invisible.

The tip used in this series is a serrated aggressive knife and the Superlong Payner 360° shape designed for bone fragmentation and removal [29] (Figures 1(d) and 2(a)).

### 2.3. Surgical Technique

The procedure was performed with the patient in a supine position under general anesthesia. A single dose of IV dexamethasone (8 mg) and IV cefazolin (1 g) were given during surgery. After corneal lubrication, the patient was prepped and draped in a sterile fashion.

The marked triangle incision was incised (Figure 1(a)), and an initial lateral canthotomy was made in a “crow's foot” using a no. 15 Bard-Parker® surgical blade (Becton Dickinson, Hancock, NY, USA). Dissection was performed in the preseptal plane to provide wide exposure of the rim of the lateral orbital wall (Figure 1(b)). The periosteum was incised using a needle-tip monopolar electrocautery, and the lateral wall was completely exposed by cutting cautery and periosteal elevators (Figure 1(c)). The posterior leaf of the periosteum was mobilized and reflected, along with the temporalis muscle; this minimizes damage to the temporalis muscle during surgery and reduces future temporal hollowing.

A protective 18 mm malleable retractor was inserted alongside the inner surface of the lateral wall, and an ultrasonic serrated aggressive knife was used to make a full-thickness cut 5 mm posterior and parallel to the lateral orbital rim, from the level of the orbital roof to the floor (Figure 1(e)). The serrated knife is used to create the superior and inferior lateral partial wall osteotomies with the power settings between 80 and 100%, the aspiration settings at 80% and maximum irrigation. The partial lateral rim was fractured out. This bone was then freed using a hammer and chisel and removed en bloc for better visualization and easier access to the deep orbit (Figure 2(b)). The cut bone was not repositioned back.

**Figure 3:**
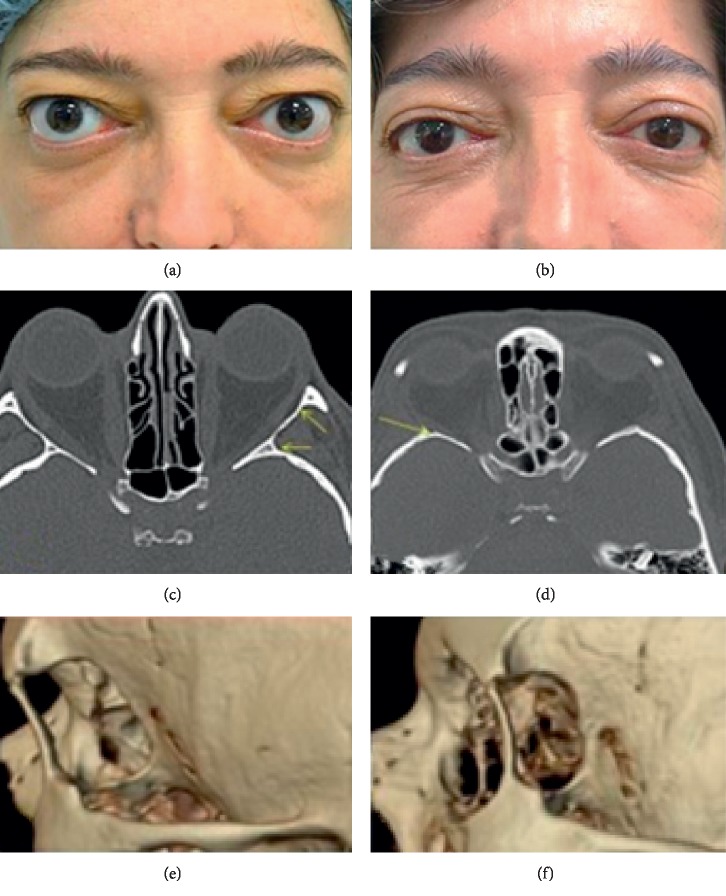
(a) Patient before surgery and (b) following decompression showing a reduction of proptosis and palpebral fissure. (c) Orbital computed tomography (CT) before surgery. The arrows show the extent of the lateral wall which will be removed to create a full-thickness bony window. (d) Postsurgery orbital CT showing a reduction of exophthalmos. It also illustrates the preservation of the orbital rim, removal of the lateral wall and sphenoidal trigone (arrow), and the soft tissue prolapse into the newly created spaces (e) and postoperative three-dimensional CT reconstruction (f).

The DLW to the trigone of the greater wing of the sphenoid was also removed using a Payner 360° ultrasonic tip (Figures 2(a) and 3(d)). The bone is relatively thin at the suture between the greater sphenoid wing and the anterior temporal bone squama (approximately 5 mm thick), and the temporal lobe dura mater can be encountered with minimal effort. Great care was taken during the drilling procedure to use gentle graded pressure, reduced potency, and short bursts of energy to minimize the risk of dura damage [19]. The dura mater was not always exposed, as the amount of bone removed from each wall correlates to the amount of orbital expansion achieved and can, therefore, be tailored to the needs of the individual patient (Figures 3(c)–3(f)) [20]. Visualization during this dissection was enhanced by the surgeon's use of a fiberoptic headlight. Electrocautery and bone wax were used to obtain hemostasis.

Once the bony removal was complete creating an adequately-sized bony window and hemostasis had been achieved, the periorbita was opened with a surgical blade or scissors in a posterior to anterior direction to allow posterolateral prolapsing of the lacrimal gland and the orbital fat into the new window (Figure 2(c)). We also opened with scissors the periorbital membrane under the lateral rectum separating the intra- and extraconal fat to allow it to prolapse. The prolapse of orbital fat in the newly opened bony spaces was encouraged with gentle manual pressure over the ocular globe. In no case, the orbital fat was excised. The incision was closed in layers with 6-0 nonabsorbable nylon suture after inserting a vacuum drain and a gentle dressing applied overnight (Figure 2(d)). Patients were kept in for 24 hours postoperatively.

After surgery, the patients were prescribed oral amoxicillin/clavulanic acid 875/125 mg twice daily and oral dexketoprofen 25 mg every 8 hours for 1 week.

#### 2.3.1. Data Collection

All patient charts were evaluated retrospectively. Data collected included demographics, diplopia (diplopia was defined as double vision within a 30-degree visual field in the primary gaze on a Hess chart) [30], visual acuity (VA) (using Snellen scale, decimal fraction), proptosis (as measured with Hertel exophthalmometer), and eyelid retraction (clinically measured by ruler—upper eyelid margin distance to the corneal reflex (MRD_1_) and lower eyelid margin distance to the corneal reflex (MRD_2_)). The presence of the following was documented: lagophthalmos, epiphora, chemosis, exposure keratopathy, zygomatic hypoaesthesia, chewing alterations, and temporal hollowing (Table 1). All measurements were taken preoperatively and on days 1, 60, and 180 postoperatively.

Statistical analysis was performed using paired sample *t* test to compare the preoperative and postoperative exophthalmometry measurements. In patients who underwent bilateral surgery, the side with the smallest difference was used to assess whether the difference between the preoperative and postoperative measurements were statistically significant.

## 3. Results

From January 2015 through September 2017, the author performed ultrasonic DLW-PRS decompression in a total of 35 patients (58 procedures, as some were bilateral). 74.2% were female (26), reflecting the female preponderance in TRO. Mean age was 52.6 ± 13.9 years. 50% of the patients were in between 48 and 61 years old, once again reflecting epidemiological data. 55.1% (32) of the procedures were carried out on the right eye, with 43.1% (25) carried out on the left. 23 patients (65.7%) had bilateral decompressions. All of the surgeries were performed for disfiguring proptosis with some degree of exposure keratopathy.

The mean preoperative exophthalmos was 24.51 ± 1.76 mm. This was reduced after surgery to 19.61 ± 1.27 mm. Hertel exophthalmometry was measured both in pre- and postsurgery with support on the orbital rim that remained preserved with this technique. The average amount of proptosis reduction was 4.9 ± 1.54 mm (range, 3–7 mm) (Figures 3(a) and 3(b)). 13 patients (37.1%) had subjective diplopia in the primary position of the gaze before surgery; 5 of these patients reported improvement or complete resolution of diplopia after surgery. Of the remaining 22 patients (62.8%), 3 (13.6%) developed immediate postoperative diplopia that in all cases resolved within 1 month. All 22 patients remained symptom free after 6 months follow-up. Preoperative MRD_1_ was 5.25 ± 0.88 mm, and the postoperative MDR_1_ was 4.49 ± 0.7 mm. MRD_2_ was 6.3 ± 0.88 mm in the preoperative setting and 5.0 ± 0.17 mm postoperatively (*p* < 0.001), Wilcoxon signed-rank test (Figures 3(a) and 3(b)).

Presence of lagophthalmos was reduced from 35 eyes (60.3%) to only five (8.6%) in the postoperative period. Presence of epiphora was also reduced from 20 patients (57.1%) to 3 (8.5%) after decompression. In 25 patients (71.4%), exposure keratopathy completely settled, and in the remaining 28.6% having a significant improvement, it completely settled after 6 months using simple lubrication. No chemosis was detected in any of our cases.

Zygomatic hypoesthesia affecting either the zygomaticotemporal or the zygomaticofacial nerves was present in the early postoperative period in 20 patients (57.1%), and in all cases, it was settled by the 6-month follow-up. 10 patients (28.5%) had chewing alterations at the early postoperative period which were also resolved by the 6 month follow-up visit. There were no other surgical complications such as cerebrospinal fluid (CSF) leak, persistent anisocoria, or accommodation deficits. We did not observe temporal hollowing in any of the patients after surgery (Table 1).

## 4. Discussion

There have been multiple descriptions of orbital decompression techniques. In 1899, Krönlein described, for the first time, a technique using lateral access to the orbit to remove orbital tumors. Dollinger used this technique for orbital decompression in exophthalmos [31].

In recent years, lateral wall bony decompression for TRO has become the first surgical option for many surgeons [12]. Recent studies [5] have shown that DLW decompression (including removal of the greater wing of the sphenoid bone) allows a bigger decompression than that obtained with the traditional lateral wall bony decompression or the paranasal sinuses decompression. This is due to the positioning of the sphenoidal greater wing behind the globe, with its removal allowing a better globe repositioning [7, 18].

In this study, we access the orbit using Nemet and Martin's lateral triangle flap technique [16]. This allows a wide exposure of the lateral orbital rim and a wider surgical field that is more centrally and inferiorly placed. Compared to other incisions used in DLW-PRS decompression such as the superior palpebral crease [20] and the canthus-sparing lateral canthotomy [5], we can achieve similar cosmetic outcomes as the incisions are placed in Langer's relaxed skin tension lines (Figure 2(e)) [16].

In our series, we partially preserve the orbital rim. We feel its removal does not help the reduction of proptosis, as shown by Zhang et al. [23]. Besides, the orbital rim plays an important role in the protection of the ocular globe; it helps define the external shape of the orbit and is an essential structure in the lateral support of the orbit in the lateral vertical maxillary buttress, which is essential in face stability [6, 13, 23, 32]. Our approach avoids the use of sutures, glue, or titanium plates used in the reattachment of the rim. This reduces the costs and the complications related to this procedure such as infections, dysaesthesia, cold intolerance, and pain [33]. Other authors [8, 34] noted temporal depression following removal of the orbital rim after DLW decompression, being less noticeable if the rim was left intact. Zhang et al. recommend using DLW-PRS decompression to minimize iatrogenic temporal depression caused by disruption of the temporal muscle [23]. As in Metha and Durrani's paper [5], there were no cases of temporal depression in our series.

We performed the DLW-PRS decompression using an ultrasonic device. There are very few papers looking at this technique. In Cho et al.'s paper, SONOPET® ultrasonic bone removal was used for DLW-PRS decompression using a temporal fossa access [5, 19, 20, 23]. Our series reports a larger number of procedures with more outcomes and longer follow-up, of at least 6 months, which is the time where maximum exophthalmos reduction is expected to be present, as well as resolution of transient diplopia [7].

Takahashi et al. [35] used SONOPET® for DLW, without complete removal of the lateral wall, removing it only to the cortical layer, as well as removing the orbital fat. However, very few details of the surgical technique were given, with only reports of proptosis reduction and chemosis 3 weeks postoperatively. Metha and Durrani reported 17 patients (21 orbital decompressions) using our same technique but creating the bony window using 90-degree bone rongeurs [5]; Chang and Piva treated 33 patients using a traditional motor, approaching the lateral orbital wall from the temporal fossa [20]. Cho et al. used the same access in 18 orbital decompressions, with an average follow-up of 70 days [19]. In all of our cases, we used an ultrasonic motor, with the aggressive knife tip, with which bone removal is accomplished with minimal manual pressure allowing us to do quick and precise thin and straight osteotomies [27, 36]. Like Cho et al., we feel this technique is advantageous due to its stability, as it eliminates the spinning motion of the high-speed drill; this can in turn cause problems such as kicking, skipping, chatter, and uncontrolled movements that can destabilize the surgeon's hand and visual obstruction of the surgical field due to bone dust. The oscillation produced by the SONOPET® only affects the tissues when they are in contact with the tip, without causing any movement of the surgeon's hand [19]. Because of the nonrotational design, soft tissues and cottonoid pledgets are not grabbed and spun by the tip, and there is little or no torque, both potentially negative features of a standard drill [24, 29]. In our experience, this results in less soft tissue damage and less torque-induced bone fragment displacement, which could lead to paralytic strabismus, anisocoria, or accommodation problems [7], protecting critical areas such as the dura mater and the neurovascular bundles in the narrow environment of the orbit. The ability to easily sculpt bone into a contoured shape with minimal bleeding and minimal postoperatory inflammation is particularly advantageous in orbital surgery [24, 27, 29]. We agree with Cho et al. that, due to these advantages, it can be safely used in tighter surgical spaces, decreasing the need to remove the lateral orbital rim [19]. The SONOPET® uses pedal-controlled simultaneous irrigation and aspiration over the surgical field, allowing one-handed use and obviating the need for separate irrigation and aspiration. This continuous cooling of the equipment prevents heat-generated damage of the soft tissues, neurovascular bundles, and bone [27, 37, 38]. This will allow a reduction of instruments in the surgical field (both inside and outside the orbit), minimizing the interruptions required to control these functions when using the traditional drill. This can, in turn, reduce the duration of the procedure [19].

Some authors [37] consider that a disadvantage of the ultrasonic technology is the relatively slow rate at which it emulsifies bone being slower than with high-speed drills. Cho used a single ultrasonic tip (not specified but likely to be the Spetzler Claw tip), for DLW decompression in 18 patients and noted it to be faster than using the reciprocating saw, rongeurs, and cutting burr on a high-speed drill in other 18 patients. We used in all cases the aggressive knife to quickly remove en bloc the orbital lateral wall, allowing also a good visualization and easy access to the deep orbit, allowing us to remove the trigone using the Payner tip safely and rapidly. We find that being able to use different tips is an advantage and feel this can contribute to reducing the surgical time, reducing the continuous energy application, especially important in bilateral cases. We feel the increased costs are compensated by the shorter surgical and anesthetic costs and should lead to lower morbidity and faster and easier patient recovery time, but further studies would be required to prove this feeling.

Our study population is comparable to previous studies, with a female preponderance and a mean age of 52.6 ± 13.9 years [5, 18, 20, 23]. The indication for surgery in all of our cases was disfiguring proptosis with ocular exposure symptoms [5, 23]. However, in our series, the majority of patients had bilateral surgery, while in other series, the surgeries were mostly unilateral [5, 23].

In our series, the preoperative exophthalmos was 24.51 ± 1.76 mm reducing to 19.61 ± 1.27 mm postoperatively. In Zhang et al.'s series, the preoperative measurements, assessed using computerized tomography, were smaller 18.7 ± 1.1 mm [23]. The measurement of exophthalmos by Hertel exophthalmometry before and after surgery is reliable. Proptosis measurements by Hertel exophthalmometry vs. computed tomography are comparable end equally effective [39]. We achieved an average of 4.9 mm of proptosis reduction, which is very similar to that achieved by other studies using DLW-PRS decompression [5, 20]. Cho et al. [19] achieved a proptosis reduction of 3.9 mm using SONOPET®, and Zhang et al. [23], using a traditional motor, achieved 3.5 mm reduction in 8 cases of DLW-PRS decompression compared to 3.6 mm in 10 cases of DLW decompression with removal of the orbital rim (Table 2).

Even though decreased retrobulbar fat volume may result in proptosis reduction [40], we did not eliminate the intraconal fat in our series, as it is a longer procedure, can take longer time to control bleeding, and has an increased risk of damaging structures within. Intraconal orbital fat carries numerous vessels and nerves that risk being injured in fat debulking surgery [30, 40, 41]. We wanted to assess how effective our surgical approach was, while at the same time minimizing iatrogenic intraoperative complications, postoperative problems, bleeding, and edema around muscles related to the disruption of intermuscular septa [42, 43]. Nevertheless, we have not found a real difference in the desired proptosis reduction when fat reduction was added to DLW decompression [42, 44] compared to simply releasing it and allowing it to expand in the osteotomy space [5].

In our study, we noted a reduction of both MRD_1_ (5.25 ± 0.88 mm preoperatively and 4.49 ± 0.70 mm postoperatively) and MRD_2_ (6.30 ± 0.88 mm preoperatively and 5.00 ± 0.17 mm postoperatively). This translated in a reduction in the vertical palpebral aperture (VPA) similar to other studies [20]. Zhang et al. [23] reported a reduction of both MRD_1_ and MRD_2_ of 1 mm. Cho et al. [19] reported a less significant result with an MRD_1_ reduction of 0.2 mm with a 0.2 mm reduction of the scleral show. Chang et al. [20] reported a 2.6 mm reduction in the VPA. We agree with other authors that the reduction of proptosis, MRD_1_, and MRD_2_ results in a reduction in the VPA and, therefore, in a reduction of the ocular surface exposure [9, 19, 20, 23, 45]. This could explain the reduction of epiphora (from 57.1% to 8.5% postoperatively), lagophthalmos (from 60.3% to 8.6%), and exposure keratopathy (from 71.5% to 28.5%) that we observed in our study.

Reducing the incidence of postoperative diplopia is an important goal in any orbital decompression, and rates of new-onset strabismus vary among surgeons and techniques, ranging from 0% to 62.5% [46, 47]. Lateral wall decompression studies share a significantly lower incidence of new-onset diplopia when compared with techniques using decompression into the paranasal sinuses due to the limited lateral shifting of the orbital contents [5, 20]. Goldberg et al. [48] reported new-onset strabismus in 7% of his patients postoperatively. In our series, we reported significant improvement or resolution of preoperative diplopia of 5 patients, with no longstanding new diplopia. These findings are similar to other studies [46, 48] that could lead to a diffuse pressure pain leading to the limitation in the eye movements. By increasing the orbital space and breaking up some of the fibrous septa within the congestive orbit, it could improve ocular motility [48]. 3 of our patients (13.6%) reported short-term diplopia immediately postoperatively, which was resolved after 1 month and remain absent at the 6-month review. Other studies also noted transient diplopia [5, 15, 20, 46, 48] (Table 2).

Transient hypoaesthesia of the zygomaticotemporal and zygomaticofacial nerves was one of the main complications noted in our series (57.1%). The incidence reported in other series was variable from 18% [5] to 100% [20], but like in our series, it was self-limiting. Chewing oscillopsia was noted in 28.5% of our cases, similar to Fayers et al.'s work with 35% [49]. Interestingly, Mehta and Durrani [5] reported no cases with the same technique. Like previous authors, we didn't encounter complications such as accommodation reflex problems or anisocoria. Some authors reported 2–7.7% CSF leaks [20, 50, 51], which increased the risk of infections such as meningitis [52]. This may occur in cases that required maximum reduction of proptosis which led to the exposure of the dura mater. For patients requiring more limited reduction, this could be achieved with a less aggressive bone reduction of the temporal fossa base, without exposing the dura mater [20]. The objective in our study was to eliminate cortical bone until dura mater was visualized, but in no case was it widely exposed [53]. Such a large reduction in proptosis is not always necessary (our mean proptosis was 24.51 ± 1.76 mm preoperatively). In cases needing significant reduction of proptosis, a medial decompression can be associated, widely exposing the dura [54]. We did not encounter any CSF leak. This could be explained by the lower propensity of the ultrasonic device to damage soft tissues and dura compared with the traditional drill [27, 36, 55]. The technique we perform with an ultrasonic device is aimed at an effective and safe reduction of moderate proptosis.

### 4.1. Limitations

This study result should be interpreted in the context of its limitations, which include its retrospective, nonrandomized nature. From a statistical standpoint, the sample size in our study is relatively small. There are only a few studies using this surgical technique to compare our results against. Larger prospective studies are required to corroborate our results and conclusions.

### 4.2. Conclusions

The described technique of DLW-PRS decompression for TRO using SONOPET® appears to be safe and effective, reducing the complications associated with decompressing the orbital floor and medial wall. The mechanical characteristics of this surgical tool (nonrotational mechanism, low profile, and directional cutting surface) provide protection to adjacent dura mater and neurovascular structures when working in narrow spaces reducing the potential risk or dura mater breach and leading to CSF leak. Advantages of this ultrasonic device include its ease of use and the ability to easily remove and sculpt bone and the reduced need to remove the lateral orbital rim.

Our findings reproduce and validate previously published papers and add new surgical modifications as well as the use of ultrasounds for this. New studies in this interesting technology will be very useful in orbital surgery.

## Figures and Tables

**Table 1:** Results.

	Preoperative	Postoperative
Exophthalmos	24.51 ± 1.76 mm	19.61 ± 1.27 mm
Visual acuity	0.8 ± 0.14	0.9 ± 0.12, *p*=0.039
Diplopia	13 (37.1%)	8 (13.7%)
MRD_1_	5.25 ± 0.88 mm	4.49 ± 0.7 mm
MRD_2_	6.3 ± 0.88 mm	5.0 ± 0.17 mm
Epiphora	20 (57.1%)	3 (8.5%)
Zigomatic hypoaesthesia	0%	40%
Lagophthalmos	60.3%	8.6%
Chewing alterations	0%	28.5%
Temporal hollowing	0%	0%

All measurements are in millimeters. Data are no. (%) unless otherwise indicated. Values are represented as mean ± standard deviation (SD); MRD_1_, upper lid margin distance to the corneal reflex; MRD_2_, lower lid margin distance to the corneal reflex.

**Table 2:** Comparison of reduction of exophthalmos and incidence of postoperative diplopia following deep lateral wall decompression.

Deep lateral wall decompression	Proptosis reduction (average, mm)	Postoperative new-onset diplopia
Ben Simon et al. [46]	3.4	2.6%
Liao et al. [15]	3.8	5.7%
Baldeschi et al. [7]	2.3	13.3%
Chang and Piva [20]	4.5	3% (transient diplopia)
Mehta and Durrani [5]	4.8	18% (transient diplopia)
Cho et al.^*∗*^ [19]	3.9	No data
Zhang et al. [23]	3.5	No data
Takahashi et al. [35]^*∗∗*^	4.8–5.3	No data
Current study^*∗*^	4.9	8.5% (transient diplopia)

^*∗*^Deep lateral wall decompression with partial rim sparing using SONOPET®. ^*∗∗*^Deep lateral wall decompression using SONOPET®.

## Data Availability

The data used to support the findings of this study are available from the corresponding author upon request.
